# Acute upregulation of neuronal mitochondrial type-1 cannabinoid receptor and it’s role in metabolic defects and neuronal apoptosis after TBI

**DOI:** 10.1186/s13041-016-0257-8

**Published:** 2016-08-02

**Authors:** Zhen Xu, Xiao-Ai Lv, Qun Dai, Yu-Qing Ge, Jie Xu

**Affiliations:** 1 Department of Neurosurgery, First affiliated Hospital of Zhejiang Chinese Medicine University, 54 Youdian Lane, Hangzhou, 310006 China; 2 Department of Surgery, First affiliated Hospital of Zhejiang Chinese Medicine University, 54 Youdian Lane, Hangzhou, 310006 China; 3 Central laboratory, First affiliated Hospital of Zhejiang Chinese Medicine University, 54 Youdian Lane, Hangzhou, 310006 China; 4 Department of Neurosurgery, Huzhou Central Hospital, 198 Hongqi Lane, Huzhou, 313003 China

**Keywords:** Apoptosis, Mitochondrial type-1 cannabinoid receptor, Metabolic defects, Traumatic brain injury

## Abstract

Metabolic defects and neuronal apoptosis initiated by traumatic brain injury (TBI) contribute to subsequent neurodegeneration. They are all regulated by mechanisms centered around mitochondrion. Type-1 cannabinoid receptor (CB1) is a G-protein coupled receptor (GPCR) enriched on neuronal plasma membrane. Recent evidences point to the substantial presence of CB1 receptors on neuronal mitochondrial outer membranes (mtCB1) and the activation of mtCB1 influences aerobic respiration via inhibiting mitochondrial cyclic adenosine monophosphate (cAMP)/protein kinase A (PKA)/complex I pathway. The expression and role of neuronal mtCB1 under TBI are unknown. Using TBI models of cultured neurons, wild type and CB1 knockout mice, we found mtCB1 quickly upregulated after TBI. Activation of mtCB1 promoted metabolic defects accompanied with ATP shortage but protected neurons from apoptosis. Selective activation of plasma membrane CB1 showed no effects on neuronal metabolism and apoptosis. Activation of mtCB1 receptors inhibited mitochondrial cAMP/PKA/complex I and resulted in exacerbated metabolic defects accompanied with a higher ratio of ATP reduction to oxygen consumption decrease as well as neuronal apoptosis. Further research found the remarkable accumulation of protein kinase B (AKT) on neuronal mitochondria following TBI and the activation of mtCB1 upregulated mitochondrial AKT/complex V activity. Upregulation of mitochondrial AKT/complex V activity showed anti-apoptosis effects and alleviated ATP shortage in metabolic defects. Taken together, we have identified mtCB1 quickly upregulate after TBI and a dual role the mtCB1 might play in metabolic defects and neuronal apoptosis initiated by TBI: the inhibition of mitochondrial cAMP/PKA/complex I aggravates metabolic defects, energy insufficiency as well as neuronal apoptosis, but the coactivation of mitochondrial AKT/complex V mitigates energy insufficiency and neuronal apoptosis.

## Introduction

Metabolic defects are common pathological processes after traumatic brain injury (TBI) and relate to subsequent brain atrophy and cognitive dysfunction [1]. Metabolic defects following TBI are not the results of ischemia or metabolic substrate insufficiency and the underlying mechanisms are not definitely clarified. TBI initiates a number of biochemical processes which lead to massive ion shifts with increased calcium in the intracellular compartment. Calcium overload in mitochondrion impairs mitochondrial function in aerobic metabolism and eventually impedes energy production even when there are sufficient oxygen and substrate present [2]. Neuronal apoptosis is another pervasive pathological phenomenon responsible for the neurodegenerative changes following TBI [3], which is also tightly associated with the mitochondrial mechanisms [4].

Endocannabinoids are implicated in a broad range of neurobiological processes including neuronal survival after cerebral ischemia or trauma [5, 6]. Endogenous cannabinoids produce the majority of their biological effects by activating two receptors: type-1 cannabinoid receptor (CB1) and type-2 cannabinoid receptor (CB2). CB1 receptors are the most abundant G-protein coupled receptor (GPCR) in mammalian brain and account for most of the biological actions of cannabinoid drugs. Neuronal CB1 receptors are the more abundant CB1 receptors which are highly enriched on neuronal plasma membranes. Although, the CB1 receptors are identified as typical plasma membrane GPCRs, recent evidences have pointed to the substantial presence of CB1 receptors on neuronal mitochondrial outer membranes and their activation influences mitochondrial cyclic adenosine monophosphate (cAMP) accumulation, protein kinase A (PKA) activity, complex I activity and mitochondrial respiration [7].

The expression and roles of neuronal mtCB1 under TBI are currently unknown. Considering the central role of mitochondria in regulating cellular aerobic metabolism and apoptosis, it is reasonable to hypothesize mtCBI might play roles in TBI-induced metabolic defects and neuronal apoptosis. Here, we investigated the early expression of neuronal mtCB1 and it’s action on metabolic defects and neuronal apoptosis following TBI by selectively using cell-permeable or impermeable CB1 receptor ligands in TBI models of cultured neurons, wild type and CB1 knockout mice.

## Results

### TBI induced acute upregulation of mtCB1 receptors

**Fig. 1:**
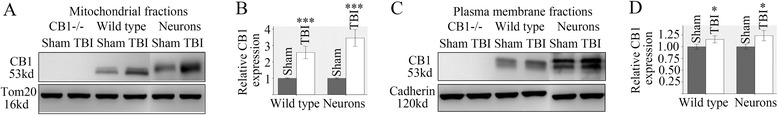
TBI induced acute upregulation of mtCB1 and plasma membrane CB1 24 h post injury. Western blot analysis of mtCB1 and plasma membrane CB1 24 h post injury showed CB1 increased in mitochondria separated from cultured neurons and wild type mice after injury but not CB1^−/−^ mice (**a**, **b**). CB1 relatively slightly increased in plasma membrane fractions separated from cultured neurons and wild type mice after injury but not CB1^−/−^ mice (**c**, **d**). Cadherin is a plasma membrane protein, Tom20 is a mitochondrial protein. Values were expressed as mean ± SE (*p < 0.05 versus sham, ***p < 0.001 versus sham)

At first, we investigated the expression of mtCB1 receptors at acute stage on ipsilateral cortex and cultured neuron post lesion. WB analysis revealed TBI caused great upregulation of CB1 receptors on mitochondria (2.5 times higher than sham in cortex of wild type mice and 3.5 times higher than sham in cultured neurons) 24 h post injury (Fig. 1a and b). Only relatively small CB1 increase was observed on plasma membranes (1.16 times higher than sham in cortex of wild type mice and 1.25 times higher than sham in cultured neurons) 24 h post injury (Fig. 1c, d). CB1 expression could hardly be detected in CB1 knockout mice (Fig. 1a, c).

### Activation of mtCB1 aggravated metabolic defects following TBI

**Fig. 2:**
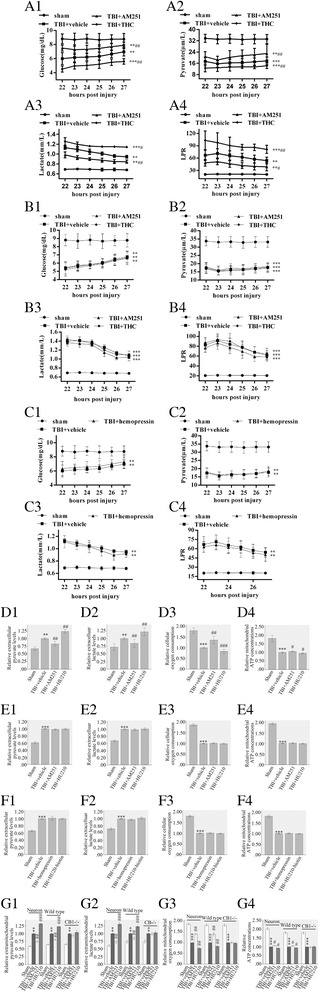
MtCB1 regulated metabolic defects following TBI. Traumatic injury changed metabolite levels in wild types, CB^−/−^ mouse brains as well as neuronal models (A1–A4, B1–B4 and D1–D4). THC and AM251 reversely mediated metabolite levels in wild type mice brain following TBI (A1–A4). THC and AM251 did not affect metabolite levels in CB^−/−^ mice brain following TBI (B1–B4). Hemopressin did not affect metabolite levels in wild type mice brain following TBI (C1–C4). Traumatic injury changed metabolite levels in cultured neuronal models (D1–D4). HU210 and AM251 reversely mediated metabolite levels in cultured neurons following injury (D1–D4). HU210 and AM251 did not changed metabolite levels in cultured CB^−/−^ neurons following injury (E1–E4). HU210-biotin and hemopressin did not affect metabolite levels in cultured neurons (F1–F4). HU210 and AM251 reversely mediated metabolite levels in mitochondria separated from cultured neurons or wild-type mice after injury, but not in mitochondria from CB1−/− mice after injury (G1-G4). Data were expressed as mean ± SE (**p < 0.01 versus sham, ***p < 0.001 versus sham, #*p* < 0.05 versus vehicle, ##*p* < 0.01 versus vehicle, ###*p* < 0.001 versus vehicle)

Next, we investigated the effects of CB1 on aerobic metabolism after TBI in vivo. The cell-permeable CB1 agonist tetrahydrocannabinol (THC, 5 mg/kg) and antagonist/inverse agonist AM251 (0.5 mg/kg) were used to control the activity of intracellular CB1 receptors. Microdialysis in wild type brains from 22 to 27 h post injury showed lower glucose and pyruvate levels, and increased lactate level as well as lactate/pyruvate ratio (LPR) (Fig. 2A1–A4). Metabolic defects were also demonstrated by the changed levels of metabolites in injured CB^−/−^ brains (Fig. 2B1–B4). It was noticeable that CB1 knockout promoted metabolic defects after TBI as demonstrated by microdialysis of metabolite levels in CB^−/−^ and wild type mice (*p* < 0.05).

In wild type mice, intraperitoneal administration of THC (5 mg/kg) resulted in decreased glucose and pyruvate levels, and increased lactate level as well as LPR suggesting an aggravated metabolic condition while AM251 showed opposite effects (Fig. 2A1–A4). Metabolite analysis was also made in CB1^−/−^ mice treated with THC (5 mg/kg) or AM251 (0.5 mg/kg) after injury and results showed metabolite levels were not significantly differ from those of vehicle (Fig. 2B1–B4) indicating the CB1 receptors were the specific targets of THC and AM251. Single inhibition of plasma membrane CB1 receptors by cell-impermeable CB1 antagonist/inverse agonist hemopressin (0.5 mg/kg) did not show any effects on aerobic metabolism after TBI suggesting the effects were due to the intracellular CB1 receptors (Fig. 2C1–C4).

To prove the effects were due to neuronal mtCB1 receptors but not CB1 receptors on other cells, we further tested the metabolites in neuronal models treated with cell-permeable CB1 ligands. In vivo after injury, as a result of the mitochondrial impairment, pyruvate no longer enters the tricarboxylic acid cycle and the need for lactate decrease. On the other hand, pyruvate and glycogen are heavily metabolized into lactate in astrocytes. Therefore, lactate accumulates and pyruvate decreases in the extracellular space, leading to the elevation of LPR [8]. However, in neuronal model of injury without astrocytes, the neuronal need for pyruvate reduces and the transformation of pyruvate into lactate greatly decreases due to the lack of astrocytes. Therefore, pyruvate and lactate both accumulate in the extracellular space [9].

Neuronal traumatic injury induced metabolic defects demonstrated by higher extracellular pyruvate and lactate levels, and reduced cellular oxygen consumption and mitochondrial ATP concentration as compared to sham group (Fig. 2D1–D4). In cultured neurons after injury, the cell-permeable CB1 agonist HU210 (0.5 μM) resulted in increased extracellular pyruvate (1.23 folds higher than vehicle) and lactate (1.22 folds higher than vehicle) levels, and reduced cellular oxygen consumption (83.3 % of vehicle) and mitochondrial ATP concentration (95.3 % of vehicle) (Fig. 2D1–D4). The AM251 (5 μM) decreased extracellular pyruvate (82.6 % of vehicle) and lactate (84.1 % of vehicle) levels, and increased cellular oxygen consumption (1.36 folds higher than vehicle) and mitochondrial ATP concentration (1.06 folds higher than vehicle) (Fig. 2D1–D4). HU210 (0.5 μM) and AM251 (5 μM) were also administrated to primary neuronal cultures prepared from CB1^−/−^ mouse (CB1^−/−^ neurons). HU210 or AM251 did not change brain metabolism in injured CB1^−/−^ neurons (Fig. 2E1–E4).

Then we selectively controlled the plasma CB1 receptors by hemopressin (5 μM) or cell-impermeable CB1 agonist HU210-biotin (0.5 μM) in cultured neurons. No changes were found in extracellular metabolite levels, oxygen consumption or mitochondrial ATP concentration after treatment (Fig. 2F1–F4).

To determine the effects were due to the direct modulation of mtCB1 or to an indirect function of CB1 receptors located on other intracellular organelles such as lysosomes [10], mitochondria were purified from injured neurons, wild type and CB1^−/−^ mice after injury then treated with HU210 (0.5 μM) or AM251 (5 μM). The drugs still apparently controlled pyruvate and lactate levels, oxygen consumption and ATP concentration in mitochondria separated from cultured neurons or wild-type mice after injury, but not in mitochondria separated from CB1^−/−^ mice after injury (Fig. 2G1–G4).

### MtCB1 activation protected neurons from apoptosis following injury

**Fig. 3:**
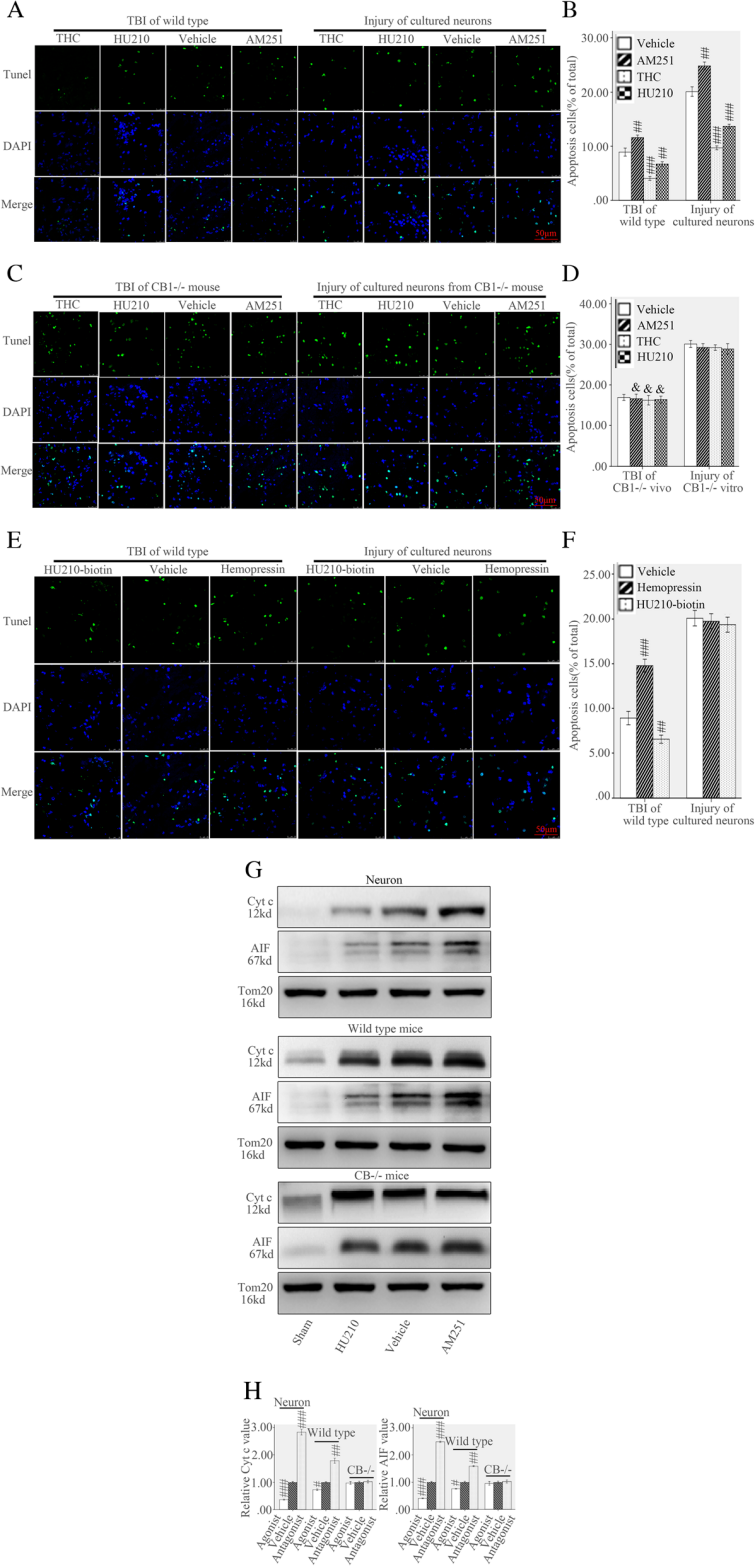
MtCB1 regulated neuronal apoptosis following injury. Both in vivo and *vitro* of wild type 24 h post injury, TUNEL study showed THC and HU210 protected neurons from apoptosis while AM251 promoted apoptosis compared with vehicle (**a**, **b**). THC, HU-210 or AM251 did not affect cellular apoptosis both in vivo and *vitro* of CB1^−/−^ (**c**, **d**). Compared with vehicle, hemopressin or HU210-biotin did not affect cellular apoptosis in vitro of wild type but reversely mediated apoptosis in vivo of wild type (**e**, **f**). Western blot analysis showed HU210 or AM251 reversely mediated mitochondrial cyt c and AIF release of wild type both in vivo and *vitro* but not in CB1^−/−^ mice (**g**, **h**). Nuclear morphology was indicated by DAPI staining and DNA breaks were detected by TUNEL analyses. Tom20 is a mitochondrial protein. bar: 25 μm. Data were expressed as mean ± SE (#*p* < 0.05 versus vehicle, ##*p* < 0.01 versus vehicle, ###*p* < 0.001 versus vehicle)

In wild type model of TBI treated with vehicle, about 8.9 % cells in vivo and 20.1 % in vitro showed apoptosis 24 h post injury (Fig. 3a, b). TUNEL study 24 h post injury showed THC (5 mg/kg in vivo and 10 μM in vitro) or HU-210 (0.1 mg/kg in vivo and 0.5 μM in vitro) significantly mitigated cellular apoptosis (4.1 % in vivo and 9.7 % in vitro in THC, 6.7 % in vivo and 13.7 % in vitro in HU-210), while the AM251 (0.5 mg/kg in vivo and 5 μM in vitro) significantly promoted apoptosis (11.6 % in vivo and 24.8 % in vitro) (Fig. 3a, b). In CB1^−/−^ models, the same dose of THC, HU-210 or AM251 did not affect cellular apoptosis both in vivo and *vitro* compared with the vehicle indicating the effects were due to CB1 receptors (Fig. 3c, d). It was noticeable that CB1 knockout promoted neuronal apoptosis both in vivo and *vitro* as demonstrated by the number of TUNEL positive cells in CB1^−/−^ and wild type treated with vehicle (*p* < 0.05) (Fig. 3a, b, c and d). In vitro of wild type hemopressin or HU210-biotin did not show any effects suggesting the neuronal plasma membrane CB1 receptors were not involved, however in vivo of wild type hemopressin promoted apoptosis and HU210-biotin inhibited apoptosis (Fig. 3e, f).

The mitochondrion serve as a crucial role in regulating neuronal apoptosis in TBI, both as an amplifier of extrinsic pro-apoptotic signal and the source of the activation of intrinsic apoptotic pathway [4]. The mitochondrial apoptotic pathway is classified as caspase dependent or caspase independent. In a caspase dependent pathway, cyt c is the necessary participant, whereas apoptosis inducing factor (AIF) is involved in the caspase independent pathway. Under pro-apoptotic stimulation such as traumatic injury, the release of cyt c from mitochondria into the cytosol triggers apoptosome assembly and the subsequent caspase activation and apoptosis, while the AIF is released and translocated into the nucleus to promote caspase independent DNA degradation [11].

To determine the effects were due to the direct modulation of mtCB1 or to an indirect function of CB1 receptors located on other intracellular organelles which could also be modulated by cell-permeable ligands, mitochondria were purified from injured neurons, wild type and CB1^−/−^ mice 24 h after injury and treated with HU210 (0.5 μM) or AM251 (5 μM). Cyt c and AIF release were measured in supernatants following pelleting of the mitochondrial suspensions. Immunoblots of supernatants revealed HU-210 significantly inhibited cyt c (36.5 % of vehicle in mitochondria of neurons and 73.2 % of vehicle in mitochondria of wild types) and AIF (41.0 % of vehicle in mitochondria of neurons and 76.0 % of vehicle in mitochondria of wild types) release, whereas AM251 promoted cyt c (2.84 folds higher than vehicle in mitochondria of neurons and 1.79 folds higher than vehicle in mitochondria of wild types) and AIF (2.49 folds higher than vehicle in mitochondria of neurons and 1.59 folds higher than vehicle in mitochondria of wild types) release (Fig. 3g, h).

### Mitochondrial cAMP/PKA/complex I inhibition promoted metabolic defects and neuronal apoptosis

**Fig. 4:**
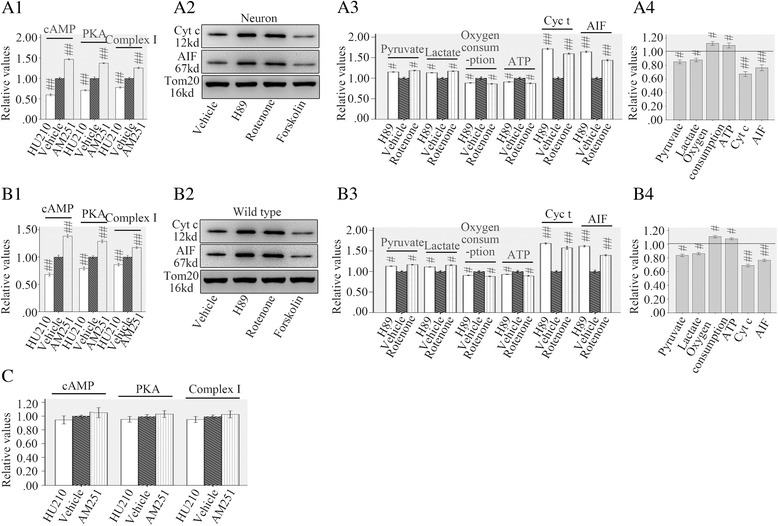
MtCB1 regulated neuronal metabolic defects and apoptosis through mitochondrial cAMP/PKA/complex I pathway. HU-210 inhibited cAMP/PKA/complex I activity in mitochondria purified from injured neurons and wild type mice while AM251 showed opposite effect (A1, B1). Mitochondrial cAMP/PKA/complex I pathway inhibitor H89 or rotenone promoted mitochondrial metabolic defects, cyt c and AIF release (A2, A3, B2 and B3) while the activator forskolin showed opposite effects (A4 and B4). HU210 and AM251 did not affect cAMP/PKA/complex I activity in mitochondria purified from CB^−/−^ mice following injury (**c**). Tom20 is a mitochondrial protein. Data were expressed as mean ± SE. (#*p* < 0.05 versus vehicle, ##*p* < 0.01 versus vehicle)

As mitochondrial cAMP/PKA/complex I pathway has been found to be the downstream target of mtCB1 and is involved in mtCB1 mediated aerobic metabolism of normal neurons [7]. We tested the possible effects of mitochondrial cAMP/PKA/complex I pathway on metabolic defects and neuronal apoptosis after TBI. Mitochondria were purified from injured neurons and wild type mice then treated with HU-210 (0.5 μM), AM251 (5 μM), H89 (a well-known pharmacological inhibitor of PKA, 1.0 mM), rotenone (specific and potent mitochondrial complex I inhibitor, 2.5 μM) or forskolin (a selective activator of adenylate cyclase, 1.5 mM). The results showed HU-210 significantly decreased mitochondrial cAMP concentration (59.7 % of vehicle in mitochondria of neurons and 68.0 % of vehicle in mitochondria of wild types), PKA activity (70.8 % of vehicle in mitochondria of neurons and 79.2 % of vehicle in mitochondria of wild types) and complex I activity (77.8 % of vehicle in mitochondria of neurons and 86.2 % of vehicle in mitochondria of wild types), while AM251 showed opposite effects on them (1.47 folds higher than vehicle in mitochondria of neurons and 1.38 folds higher than vehicle in mitochondria of wild types to cAMP, 1.37 folds higher than vehicle in mitochondria of neurons and 1.29 folds higher than vehicle in mitochondria of wild types to PKA, 1.26 flods higher than vehicle in mitochondria of neurons and 1.17 folds higher than vehicle in mitochondria of wild types to complex I) (Fig. 4A1, B1).

The inhibition of mitochondrial cAMP/PKA/complex I activity by H89 significantly increased extramitochondrial pyruvate (1.15 folds higher than vehicle in mitochondria of neurons and 1.13 folds higher than vehicle in mitochondria of wild types) and lactate levels (1.13 folds higher than vehicle in mitochondria of neurons and 1.11 folds higher than vehicle in mitochondria of wild types), and decreased oxygen consumption (88.5 % of vehicle in mitochondria of neurons and 90.5 % of vehicle in mitochondria of wild types) accompanied with ATP insufficiency (90.8 % of vehicle in mitochondria of neurons and 93.0 % of vehicle in mitochondria of wild types) (Fig. 4A3, B3). The ratio of ATP decrease to oxygen consumption reduction (percentage of ATP reduction/percentage of oxygen consumption reduction = 12.7 %/13.8 % in mitochondria of cultured neurons, 7.0 %/9.5 % in mitochondria of wild type mice) is higher than that of mtCB1 activation (percentage of ATP reduction/percentage of oxygen consumption reduction = 4.7 %/16.7 % in cultured neurons, 7.2 %/27.2 % in mitochondria of cultured neurons, 5.3 %/16.2 % in mitochondria of wild type mice) (*p* < 0.05). Parallel supernatant cyt c and AIF analysis demonstrated a significant promotion of mitochondrial cyt c (1.71 folds higher than vehicle in mitochondria of neurons and 1.68 folds higher than vehicle in mitochondria of wild types) and AIF (1.64 folds higher than vehicle in mitochondria of neurons and 1.61 folds higher than vehicle in mitochondria of wild types) release was induced by H89 (1.0 mM) treatment (Fig. 4A2, A3, B2 and B3). Similar results (1.19 folds higher than vehicle to pyruvate, 1.17 folds higher than vehicle to lactate, 86.2 % of vehicle to oxygen consumption, 87.3 % of vehicle to ATP production, 1.60 folds higher than vehicle to cyt c and 1.44 folds higher than vehicle to AIF in mitochondria of neurons/1.17 folds higher than vehicle to pyruvate, 1.15 folds higher than vehicle to lactate, 88.2 % of vehicle to oxygen consumption, 89.3 % of vehicle to ATP production, 1.58 folds higher than vehicle to cyt c and 1.40 folds higher than vehicle to AIF in mitochondria of wild types) were also seen in rotenone treatment groups (Fig. 4A2, A3, B2 and B3). The forskolin treatment showed opposite effects in mitochondria separated from cultured neurons and wild type mice (Fig. 4 A4, B4). Same dose of HU-210 and AM251 were also administrated to mitochondria separated from CB1^−/−^ mice. Results showed no changes were found in mitochondrial cAMP/PKA/complex activity suggesting CB1 receptors were the specific targets (Fig. 4c).

### Protein kinase B (AKT) accumulated in neuronal mitochondria after TBI and mtCB1 activation upregulated mitochondrial AKT/complex V activity

The direct downregulation of mitochondrial cAMP/PKA/complex I resulted in a lower efficiency of ATP synthesis compared with that of mtCB1 activation as demonstrated by higher ratio of ATP decrease to oxygen consumption reduction. In addition, direct mitochondrial PKA/complex I inhibition increased cyt c and AIF release which incurred neuronal apoptosis. This was opposite to the anti-apoptosis effects of direct mtCB1 activation as demonstrated in our experiment. Thus we deduced that there might have another anti-apoptosis pathway involved in the mtCB1 activation.

**Fig. 5:**
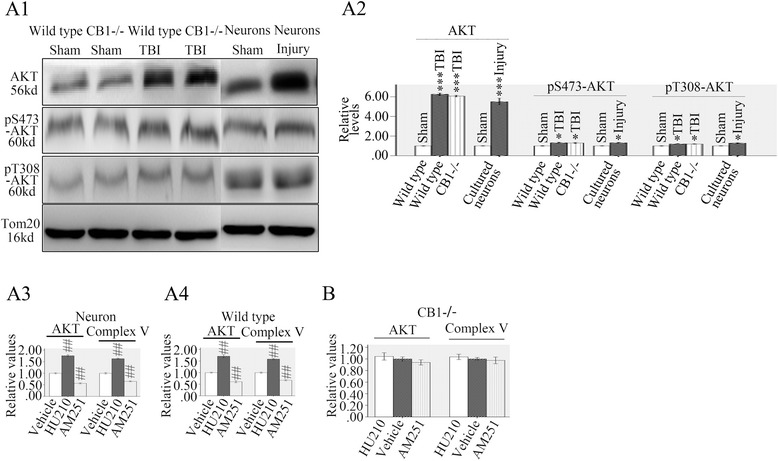
TBI induced AKT accumulation in neuronal mitochondria and mtCB1 regulated mitochondrial AKT/complex V activity. Western blot analysis of mitochondrial total AKT and pAKT showed AKT greatly increased in mitochondria separated from neurons, wild type or CB1^−/−^ mice 24 h post injury and most of them are unphosphorylated (A1 and A2). HU210 increased AKT/complex V activity in mitochondria purified from cultured neurons or wild type mice while AM251 showed opposite effects (A3 and A4). HU-210 and AM251 did not affect AKT/complex V activity in mitochondria from CB1^−/−^ mice (**b**). Tom20 is a mitochondrial protein. Data were expressed as mean ± SE. (##*p* < 0.01 versus vehicle, *p < 0.05 versus sham, ***p < 0.001 versus sham)

Protein kinase B (AKT) which is another well-known downstream target of CB1 was studied. At first, we tested whether there was mitochondrial accumulation of AKT under the stimulation of TBI. Western blots were performed on mitochondria separated from cultured neurons, wild type and CB^−/−^ mice. Western blots analysis showed approximately 5.3–6.3 folds increase to AKT protein expression in mitochondria separated from cultured neurons (5.33 folds higher than sham), wild type (6.26 folds higher than sham) and CB1^−/−^ mice (6.07 folds higher than sham) 24 h after injury (Fig. 5A1, A2). Although the AKT value in mitochondria from wild type TBI model was higher than that of CB1^−/−^ mice, the difference was statistically nonsignificant (*p* > 0.05) indicating the CB1 knockout did not influence mitochondrial AKT accumulation following TBI. Then, phospho-Ser473-AKT and phospho-Thr308-AKT, two main kind of phosphorylated AKT, were analyzed and results showed only a minor increase of phosphorylated AKT (1.32, 1.29 and 1.32 folds higher than sham in mitochondria of wild types, CB^−/−^ and neurons respectively to pSer473-AKT/1.20, 1.19 and 1.29 folds higher than sham in mitochondria of wild types, CB^−/−^ and neurons respectively to pThr308-AKT) was found on mitochondria suggesting the TBI-induced AKT accumulation on mitochondria might largely be unphosphorylated (Fig. 5A1).

Then we tested the effects of mtCB1 activation on mitochondrial AKT and complex V activity. The results showed HU-210 (0.5 μM) significantly increased mitochondrial AKT (1.76 folds higher than vehicle in mitochondria of neurons and 1.71 folds higher than vehicle in mitochondria of wild type) and complex V (1.63 folds higher than vehicle in mitochondria of neurons and 1.59 higher than vehicle in mitochondria of wild types) activities, while AM251 (5 μM) showed opposite effects (56.3 % of vehicle in mitochondria of neurons and 60.8 % of vehicle in mitochondria of wild type to AKT/65.0 % of vehicle in mitochondria of neurons and 67.2 % of vehicle in mitochondria of wild types to complex V) on them (Fig. 5A3, A4). Same dose of HU-210 and AM251 did not show any effects on mitochondria from CB1^−/−^ mice indicating the effects were due to CB1 reporters (Fig. 5b).

### Mitochondrial AKT activation alleviated neuronal metabolic defects and apoptosis

Then we investigated if the activation of mitochondrial AKT/complex V was directly involved in the protection effects on metabolic defects and apoptosis after TBI. Mitochondria were purified from injured neurons or wild type mice then treated with sodium valproate (VPA, 1.0 mM) which could increase the activation dependent phosphorylation of Ser-473 of AKT through inhibitory effect on histone deacetylase [12].

**Fig. 6:**
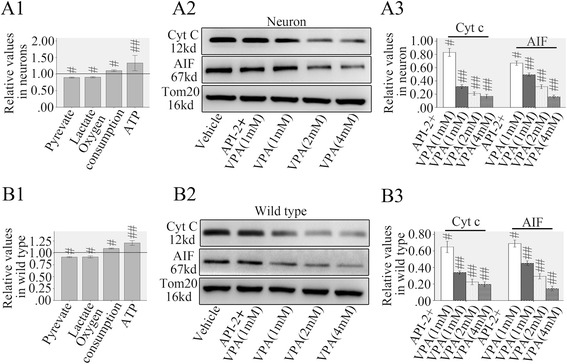
Mitochondrial AKT/complex V pathway regulated neuronal metabolic defects and apoptosis. AKT activator VPA alleviated metabolic defects, cyt c and AIF release in mitochondria purified from injured neurons or wild type mice (A1 and B1). Preincubation 30 min with AKT specific inhibitor API-2 counteracted the VPA induced cyt c and AIF release and a concentration-dependent effect was induced by 2.0 and 4.0 mM VPA (A2, A3, B2 and B3). Tom20 is a mitochondrial protein. Data were expressed as mean ± SE. (#*p* < 0.05 versus vehicle, ##*p* < 0.01 versus vehicle)

The results showed VPA resulted in decreased pyruvate (89.5 % of vehicle in mitochondria of neurons and 91.2 % of vehicle in mitochondria of wild types) and lactate (90.3 % of vehicle in mitochondria of neurons and 91.5 % of vehicle in mitochondria of wild types), and increased oxygen consumption (1.10 folds higher than vehicle in mitochondria of neurons and 1.09 folds higher than vehicle in mitochondria of wild types) accompanied with raised ATP supply (1.33 folds higher than vehicle in mitochondria of neurons and 1.21 folds higher than vehicle in mitochondria of wild types) (Fig. 6A1, B1). The ratio of ATP rise to oxygen consumption increase (percentage of ATP increase/percentage of oxygen consumption increase = 133 %/110 % in mitochondria from neuron, 121 %/109 % in mitochondria from wild type mice) was higher than that of mtCB1 activation. Parallel supernatants cyt c and AIF analysis demonstrated VPA significantly inhibited the mitochondrial cyt c (31.2 % of vehicle in mitochondria of neurons and 34.0 % of vehicle in mitochondria of wild types) and AIF (49.3 % of vehicle in mitochondria of neurons and 45.2 % of vehicle in mitochondria of wild types) release (Fig. 6A2, A3, B2 and B3). Preincubation 30 min with AKT specific inhibitor API-2 (1 mM) significantly counteracted the VPA (1 mM) induced cyt c (83.0 % of vehicle in mitochondria of neurons and 64.8 % of vehicle in mitochondria of wild types) and AIF release (66.8 % of vehicle in mitochondria of neurons and 69.0 % of vehicle in mitochondria of wild types) (Fig. 6A2, A3, B2 and B3). Examination of the concentration dependent effects revealed that greater responses were induced by 2.0 and 4.0 mM VPA (Fig. 6A2, A3, B2 and B3).

## Discussion

This is the first study to report on the effects of mtCB1 in TBI. Our results present clear evidence that at least two pathways are the downstream targets of mtCB1 and play a dual role in metabolic defects and neuronal apoptosis after TBI.

As the CB1 receptors traditionally are believed to exist on the plasma membranes, neuroprotective effects of the lipophilic cannabinoids are ascribed to nonspecific alterations of membrane properties induced by plasma membrane CB1 receptors. This study showed a relatively small upregulation of plasma membrane CB1 receptors compared to the strong upregulation of mitochondrial CB1 receptors at the first 24 h after TBI. Considering the fast and great increase of endogenous cannabinoid levels (10-fold within 4 h) following TBI [5] there might have a greater over activation of mtCB1 receptors at least at the acute stage following TBI. Consequently we further demonstrated mtCB1 activation promoted metabolic defects accompanied with ATP shortage but protected neurons from apoptosis. Although in vitro single modulation of CB1 receptors located on neuronal plasma membranes by cell-impermeable ligands did not show significant effects, in vivo inhibition of plasma membrane CB1 receptors by cell-impermeable antagonist promoted apoptosis and cell-impermeable agonist inhibited apoptosis. The underlying cause of this difference between cultured neurons and mice treated with cell-impermeable ligands was not definitely clarified in this experiment, however it was not entirely unexpected. According to previous reports, except for neurons, CB1 receptors also express on the cerebromicrovascular endothelial cells which represent the main component of the blood brain barrier and are involved in endocannabinoid (eCB) system mediated vasodilation, astrocytes and microglias all of which are main sources of inflammatory mediators following TBI [13–15]. In vivo studies have proved activation of plasma membrane CB1 receptors displays neuroprotective effect based on the multipotent properties such as the suppression of the formation of reactive oxygen species (ROS), vasodilators, anti-inflammatory agents and inhibitors of excitotoxicity [16].

The CB2 receptors which are well recognized on resident inflammatory cells within the CNS [17] and show neuroprotective effects through attenuating inflammatory response [18] might also be activated by the cell-impermeable CB1 agonist, as most CB1 agonists show little selectivity between the CB1 and CB2 receptors, thus involved in the agonist-induced protective effects in vivo. However, the contrary effects induced by the cell-impermeable CB1 antagonist could demonstrate the CB1 specificity as the CB1 antagonist compounds including hemopressin and AM251 are highly selective (>1000 fold selective for CB1 vs CB2) [19]. Thus the anti-apoptosis effect by cell-impermeable CB1 agonist in vivo might be due to the activation of plasma membrane CB1 receptors located on non-neuronal cells in brain.

For further exploring the mechanisms underlying the mtCB1 modulated aerobic metabolism and apoptosis, mitochondrial cAMP/PKA/complex I pathway was examined in mitochondria after treating with CB1 ligands. We did find mtCB1 activation inhibited mitochondrial cAMP/PKA/complex I signaling pathway and direct cAMP/PKA/complex I inhibition by CB1 agonist did aggravate metabolic defects as mentioned by Bénard [7]. However, cAMP/PKA/complex I inhibition also promoted mitochondrial cyt c and AIF release, implying a pro-apoptosis effect. This phenomenon is also consistent with the common sense that the insufficient energy supply aggravates mitochondrial impairment induced by TBI due to the failure of energy dependent membrane ion pumps to facilitate restoration of ionic gradients, thus, promotes apoptosis. It was paradoxical that mtCB1 activation via mitochondrial cAMP/PKA/complex I pathway inhibition aggravated metabolic defects accompanied with ATP insufficiency but protected neurons from apoptosis following TBI. In addition, the ratio of ATP decrease to oxygen consumption reduction after direct cAMP/PKA/complex I inhibition by H89 or rotenone was higher than that of indirect inhibition by CB1 agonist indicating mtCB1 activation might have mitigated ATP uncoupling in oxidative phosphorylation. Decrease in the coupling of the electron transport chain and oxidative phosphorylation followed by lower ATP production has been demonstrated shortly after TBI in rodents [20].

AKT (also known as PKB or RAC-PK) is an intracellular serine/threonine kinase involved in regulating cell survival. Under some stimuli, including growth factors, hormones, and stressors, AKT translocates into mitochondria and the activation of mitochondrial AKT promotes mitochondrial complex V activity which improves ATP synthesis under the dysregulation of mitochondrial oxidative phosphorylation and inhibits apoptosis in several types of cells [21–23]. A significant body of works show that activation of AKT promotes cell survival in multiple in vitro and in vivo models where neuronal death is seen, including TBI [24, 25]. The great accumulation of AKT on mitochondria shortly after injury suggested AKT activation might play a role in TBI. The mechanisms for mitochondrial accumulation of AKT have not been thoroughly investigated. Stimulation of a variety of cell types with insulin-like growth factor-1 (IGF-1), insulin, or stress (induced by heat shock), induces translocation of AKT to the mitochondria within only several minutes of stimulation, causing increases of nearly 8 to 12 folds, and the mitochondrial AKT is found to reside in the matrix and the inner and outer membranes [23]. Although not in the nervous system, AKT overexpression by a mitochondria targeted constitutively active AKT via adenoviral vector inhibits efflux of cyt c and AIF from mitochondria to cytosol and partially prevents loss of mitochondria cross membrane electrochemical gradient, showing anti-apoptosis effect [22]. As a large body of studies have revealed the early and sustained increase in the expression of IGF-1 and heat shock protein in brain following traumatic injury [26, 27], a similar mechanism involved in IGF-1 or heat shock protein induced mitochondrial AKT accumulation might also be triggered by TBI. The cytoplasmic AKT is activated when phosphorylated by G-protein βγ subunits of plasma membrane CB1 receptor [28]. The presence of AKT in the intermembrane space, inner membrane, outer membrane and matrix implies that it can connect the mitochondrial CB1 and be phosphorylated.

The F1 portion of complex V anchors on the inner membrane and protrudes into the matrix [29] and the β-subunit of F1 portion which is the catalytic site for ATP synthesis is one of the mitochondrial AKT’s substrates [23]. Mitochondrial activation of AKT increases complex V activity by 24 % in normal myocardium in vivo [21]. Through increasing complex V activity, the TBI triggered accumulation and mtCB1 induced activation of AKT in neuronal mitochondria might improve the coupling of the electron transport chain and oxidative phosphorylation as indicated by the higher ratio of ATP rise to oxygen consumption increase in this study.

The upregulated complex V activity might play an indirect anti-apoptosis role because of the subsequently increased energy supply. However the net supply of ATP was still decreased following mtCB1 activation and the anti-apoptosis effect of mtCB1 activation might not totally depend on the improved energy supply. A number of downstream targets of cytoplasmic AKT, such as CREB, FoxO transcription factors and GSK3β, have also been found on mitochondria [23, 30–32] and some of them have been proved to modulate apoptosis through a mitochondrial way [30]. A deeper investigation should be necessary for determining the downstream mechanisms of the mtCB1/AKT modulated neuronal apoptosis following TBI.

Totally, these data suggest a dual role the activation of mtCB1 receptors might play in TBI. The cAMP/PKA/complex I inhibition aggravates metabolic defects, energy insufficiency as well as neuronal apoptosis but the coactivation of AKT/complex V mitigates energy insufficiency and neuronal apoptosis. The discovery that mitochondrial CB1 signaling is involved in metabolic defects and neuronal apoptosis following TBI further extends the range of mechanisms through which endocannabinoid signaling protect brain from TBI and would possibly add new pharmacological targets for the therapeutic exploitation in endocannabinoid system.

## Methods

### Animals and TBI procedure

Experimental animal was C57BL/6 J mouse, wild-type (CB1^+/+^) afforded by animal experimental center of Zhejiang Chinese Medicine University and homozygous CB1 knockout mice (CB1^−/−^) [33]. The use and care of animals employed in our models were approved by the Animal Care and Use Committee of Zhejiang Chinese Medicine University, in accordance with all relevant laws of china. Adult male CB1^−/−^ and CB1^+/+^ littermates (10–16 weeks) were housed individually with an inverse 12/12 h light/dark cycle for at least 2 weeks before experiment. Mice (*n* = 5 per group) were suffered to TBI using a controlled cortical impact (CCI) device (VCU Biomedical Engineering Facility, Richmond, Virginia, USA) after anesthetized with isoflurane evaporated in a gas mixture containing 70 % N_2_O and 30 % O_2_. Briefly, a craniotomy of 5-mm diameter was performed over the right parietal cortex which centered around 2.0 mm posterior to bregma and 2.0 mm lateral to the midline. Injury was performed using a 3.0 mm rounded metal tip that was aimed vertically to the brain surface. The metal tip hit the brain at a speed of 4.5 m/s resulting a deformation depth of 2.0 mm. Sham injured mice underwent identical anesthesia and craniotomy procedure without CCI injury. Mice were placed for 2 h in an incubator heated to 33 °C and at a humidity of 35 % to recover in their individual cages.

### Cell culture and injury procedure

Primary hippocampal neuron cultures were prepared from 14-day wild type CB1^+/+^ and CB1^−/−^ C57BL/6 J mouse embryos as previously described and injured using an established in vitro model for TBI as described [34, 35]. The purity of cultured neurons was proved by immunofluorescence with antibody targeted to GFAP or IBA-1. Cells were suffered to severe injury and the control wells were contained in the same FlexPlates as injured wells, and thus underwent the same manipulations, except not received the deformation of the silastic membrane.

### Synthesis of HU210-biotin and drug administration

The cell-impermeable biotinylated version of the lipophilic CB1 receptor agonist HU210 (HU210-biotin) displaying a comparable affinity for CB1 receptors to that of HU210 was used as plasma membrane CB1 receptor activator and was synthesized as previous described [7]. Purity (elemental analysis) >95 %. Blood brain barrier permeability of HU-210 in mouse (intraperitoneally, 0.1 mg per kilogram) was proved by Nguyen VH [36]. Cell-impermeable antagonist hemopressin, cell-permeable lipophilic antagonist AM251 and agonist THC were used in this experiment [7]. Intraperitoneal use of THC (5 mg per kilogram), hemopressin (0.5 mg per kilogram) and AM251 (0.5 mg per kilogram) to rodent were based on previous studies [7, 37]. Injections were made in a volume of 2 ml/kg body weight and the first dose administrated 30 min after injury and repeated 12 h after injury. Drugs were added directly into cultured neurons to reach the concentrations (5 μM hemopressin, 0.5 μM of HU210, 0.5 μM HU-biot, 10 μM THC and 5 μM AM251) 30 min after injury and repeated 12 h later to keep the same concentrations without apparent cellular toxicity. In purified mitochondria, Drugs were added right after extraction and 30 min before testing with the concentration same to neuron. H89 (1 mM), rotenone (2.5 μM), forskolin (1.5 mM), valproic acid (VPA, 1.0 mM, 2.0 mM and 4.0 mM) and API-2 (1.0 mM) were also directly added into purified mitochondria. Pharmaceutical chemicals were purchased from Sigma-Aldrich.

### Cell fraction isolation

Cortex from the ipsilateral injury site was rapidly excised and cut into small pieces. Tissues or cells were homogenized in isolation buffer (1 mM EDTA and DNAase and protease inhibitor cocktail, PH7.4). The homogenate was centrifuged at 1 000 g for 5 min. The supernatant was strained through gauze and recentrifuged at 7 000 g for 10 min. The resulting pellets were resuspended in ice cold isolation buffer for a new series of centrifugation (1 000 and 7 000 g) for mitochondrial purification. Then the supernatant was collected for centrifugation for membrane fraction collection. A discontinues sucrose gradient (1.0–2.5 M) was used to layer the plasma membrane fractions. The crude mitochondrial pellets were resuspended in ficoll medium and centrifuged for further purification. Protein concentration was determined by the BCA assay. The mitochondria were made up to a concentration of 50 mg protein/ ml in the buffer. The lysosome, cytosol and endoplasmic reticulum (ER) contaminations in purified mitochondria were investigated using antibodies against LAMP1, GAPDH and calreticulin respectively. Few lysosome, cytosol and ER contaminations were seen in the purified mitochondria (data were not shown).

### Western blots

All samples were supplemented with proteases inhibitor (Roche) then diluted in sample buffer. Samples were supplemented with 2 % beta-mercaptoethanol and 50 mM DTT and boiled for about 6 min. Proteins were separated on tris-glycine 4–15 % acrylamide gels and transferred to PVDF membranes. Membranes were soaked in 5 % milk in PBS-Tween 20 (0.05–0.15 %). Mitochondrial and plasma membrane proteins were detected using rabbit anti-Tom-20 polyclonal antibody (Santa Cruz, 1:200) and rabbit anti-cadherin polyclonal antibody (Santa Cruz, 1:200). Lysosome, cytosol and endoplasmic reticulum(ER) contaminations were investigated using goat anti-LAMP1 polyclonal antibody (Santa Cruz, 1:200), goat anti-GAPDH polyclonal antibody (Santa Cruz, 1:200) and rabbit anti-calreticulin polyclonal antibody (Invitrogen, 1:300) respectively. The presence of the CB1 receptors in mitochondria and plasma membrane fractions was analyzed using rabbit anti-CB1 polyclonal antibody directed against the C terminus of the receptor (Cayman, 1:300). The mitochondrial total AKT, pS473-AKT and pT308-AKT were tested with rabbit anti-AKT polyclonal antibody (Santa Cruz, 1:300), rabbit anti-pS473-AKT polyclonal antibody (Santa Cruz, 1:300), rabbit anti-pT308-AKT polyclonal antibody (Santa Cruz, 1:300) respectively. AIF and cyt c were analyzed with rabbit anti-AIF polyclonal antibody (Santa Cruz, 1:500) and goat anti-cyt c polyclonal antibody (Santa Cruz, 1:1000). GFAP and IBA-1 were detected with rabbit anti-GFAP polyclonal antibody (Santa Cruz, 1:500) and rabbit anti- IBA-1 polyclonal antibody (Santa Cruz, 1:500). Immunoreactivity was detected by incubation with secondary HRP-coupled antibody for 1 h at room temperature followed by the ECL plus reagent (Santa Cruz). The optical densities of the bands were calculated using a MiVnt image analysis system (Bio-Rad, Carlsbad, CA, USA).

### Microdialysis

Microdialysis study was initiated at 22 h post injury and maintained for 6 h. A microdialysis catheter (CMA 12, CMA Microdialysis, Sweden) was introduced through a guide cannula secured in a dedicated and distinct burr hole placed 2 mm anteriorly to the craniectomy. Perfusion was then initiated at 1 μL/min with CNS perfusion fluid (CMA Microdialysis, Sweden) and microdialysis samples collected every hour. Concentrations of glucose, pyruvate and lactate were measured using high performance liquid chromatography (CMA 600, CMA Microdialysis, Sweden) and averaged hourly for statistical analysis.

### Measurement of extracellular lactate and pyruvate concentrations

Twenty-four hours after injury, the growth medium in six-well FlexPlates were collected, cells removed by centrifugation at 1200 g for 5 min, then the lactate and pyruvate concentrations were determined enzymatically using Biovision assay kits according to instructions.

### Oxygen consumption assay

Oxygen consumption was monitored at 30 °C in a thermostatically controlled glass chamber equipped with a clark oxygen electrode (Hansatech Instruments, Norfolk, England). Mitochondria purified from aliquots of cells were suspended in 1 mL of the respiration buffer (75 mM mannitol, 25 mM sucrose, 10 mM KCl, 10 mM Tris–HCl, 450 mM EDTA) in the chamber. Intact cells were transferred directly in the chamber. Respiratory substrates (10 mM pyruvate and 5 mM malate) were added directly to the chamber. A coupled respiratory state was obtained by adding 2 mM ADP.

### Assay for ATP

ATP assay kits (Promega) were used 24 h after injury. Aliquots of isolated mitochondria were digested with 2.5 % TCA and then neutralized with tris-acetate. The luminescence was measured using synergy HT multi-detection microplate reader. A 2 s delay time after 100 μL rLuciferase/Luciferin reagent injection and a 10s RLU signal integration time were used according to the manual.

### TUNEL staining

Consecutive coronal sections were cut from posterior bregma-1 mm to bregma-2.5 mm with 150 μm intervals. The thickness of every section was 8 μm and a total number of 10 sections in each brain were collected for terminal deoxynucleotidyl transferase-mediated dUTP-biotin nick end labeling (TUNEL) staining. TUNEL staining was performed using an *In Situ* Cell Death Detection Kit (Roche Diagnostics, Mannheim, Germany) to detect *in situ* DNA fragmentation. DAPI staining was performed according to routine laboratory methods. The images were viewed on an EVOS-fl digital inverted fluorescent microscopy.

In each section, six non-overlapped vision fields were randomly selected from regions surrounding the primary contused site. The apoptotic index of each section was calculated by the mean number of TUNEL/DAPI double positive nuclei in the six views and the apoptotic index for each animal was determined by the final average percentage of double positive cells of the 10 sections.

Cell apoptosis was assessed in cultured neurons according to previous study [38]. Neurons in six contiguous images were counted and averaged per well. All these images were taken from the center portion of the well, as this region was previously shown to receive equal impact from the cell injury controller [35]. All cell counting was performed blind.

### Detection of cyt c and AIF release

Isolated neuronal mitochondria were incubated at 30 °C for 50–80 min in 100 μL of KCl medium(125 mM KCl, 2 mM K2HPO4, 20 mM HEPES) supplemented with 5 mM succinate and 4 mM MgCl_2_. Mitochondrial suspensions were centrifuged at 13 000 g for 6 min at 4 °C at the end of the incubation period. Supernatants were stored with Halt Protease Inhibitor Mixture® (Pierce) at −80 °C. Pellets were also stored at −80 °C then resuspended in 100 μL of KCl medium containing 1 % triton X-100 and Halt Protease Inhibitor Mixture® prior to gel loading. Proteins retained in the mitochondrial pellets or released into the supernatants were separated by SDS-PAGE. AIF and cyt c were then detected by immunoblot.

### Cyclic AMP, PKA and AKT activity assay

Cyclic AMP level, PKA and AKT activity were assayed on isolated mitochondria using the Direct Correlate-EIA cAMP kit (Assay Designs Inc) and an ELISA kit (Enzo Life Science) respectively.

### NADH-ubiquinone oxidoreductase (complex I) activity assay

We followed the reaction of NADH oxidation into NAD+ by complex I on isolated mitochondria as previously described [39].

### Oxidative phosphorylation Complex V activity assay

Equal amount of mitochondrial protein was added to 800 μL pre-warmed distilled water and 200 μL prewarmed reaction buffer containing 50 mM Tris–HCl, 1 mM NADH, 5 mg/mL BSA, 20 mM MgCl_2_, 50 mM KCl, 15 μM carbonyl cyanide m-chlorophenyl hydrazone, 10 mM phosphoenol pyruvate, 5 μM antimycin and 4U of lactate dehydrogenase/pyruvate kinase at 37 °C. The activity was measured by the absorbance at 340 nm for 3 min. Twelve μM oligomycin was added to the reaction mixture to determine the oligomycin-sensitive complex V activity. The abundance of complex V subunits was analyzed with complex V immunocapture Kit (Mitosciences).

### Statistical analyses

All graphs and statistical analyses were performed using IBM SPSS software (version 21.0). Results are expressed as means of independent data points ± SE. Data were analyzed using a paired or unpaired Student’s *t*-test, one-way ANOVA (followed by Newman-Keuls post hoctest), or two-way ANOVA (followed by Bonferroni’s post hoctest).
